# DIA mass spectrometry characterizes urinary proteomics in neonatal and adult donkeys

**DOI:** 10.1038/s41598-022-27245-0

**Published:** 2022-12-30

**Authors:** Feng Yu, Yifan Chen, Bo Liu, Tao Wang, Zhaoliang Ding, Ziwen Yi, Yiping Zhu, Jing Li

**Affiliations:** 1 Equine Clinical Diagnostic Center, College of Veterinary Medicine, China Agricultural University, No. 2 Yuanmingyuan West Road, Beijing, 100093 China; 2 Dong-E-E-Jiao Co., Ltd., Dong-E County, 252299 Shandong China

**Keywords:** Computational biology and bioinformatics, Immunology, Biomarkers

## Abstract

Health monitoring is critical for newborn animals due to their vulnerability to diseases. Urine can be not only a useful and non-invasive tool (free-catch samples) to reflect the physiological status of animals but also to help monitor the progression of diseases. Proteomics involves the study of the whole complement of proteins and peptides, including structure, quantities, functions, variations and interactions. In this study, urinary proteomics of neonatal donkeys were characterized and compared to the profiles of adult donkeys to provide a reference database for healthy neonatal donkeys. The urine samples were collected from male neonatal donkeys on their sixth to tenth days of life (group N) and male adult donkeys aging 4–6 years old (group A). Library-free data-independent acquisition (direct DIA) mass spectrometry-based proteomics were applied to analyze the urinary protein profiles. Total 2179 urinary proteins were identified, and 411 proteins were differentially expressed (*P* < 0.05) between the two groups. 104 proteins were exclusively expressed in group N including alpha fetoprotein (AFP), peptidase-mitochondrial processing data unit (PMPCB), and upper zone of growth plate and cartilage matrix associated (UCMA), which might be used to monitor the health status of neonatal donkeys. In functional analysis, some differentially expressed proteins were identified related to immune system pathways, which might provide more insight in the immature immunity of neonatal donkeys. To the best of our knowledge, this is the first time to report donkey urinary proteome and our results might provide reference for urinary biomarker discovery used to monitor and evaluate health status of neonatal donkeys.

## Introduction

Urine is a biological diagnostic material which can be obtained noninvasively by free flow in large quantities. So far, there has been no study reported the value of any urine tests in donkeys, although urinalysis is frequently used on the diagnosis of urinary tract diseases and many systemic disorders in other domestic animals^1^. Mass spectrometric analysis such as proteomic has been developed for analysis of proteins at a large scale to help study different states of physiology and diagnose broader range of diseases^2^. Urinary proteins are filtered through plasma, therefore, urinary proteomics is able to provide comprehensive readout of local physiology (from the kidney-related proteins) and the systemic physiology (from the serum proteins)^3^.

The establishment of normal urinary protein profiles is critical for further investigation of diagnosing and monitoring the progression of diseases^4^. There has been normal urinary proteomics investigated in many species including human^3^, cattle^5^ and horses^6^. In healthy cows, 1564 urinary proteins in total are identified and the majority proteins are categorized into cytoplastic, catalytic activity and metabolism^5^. Urinary protein map of neonatal calves has also been studied, and unique proteins associated with kidney development and renal functions are observed^7^. These normal urinary proteomes are the basis for the identification and selection of important biomarkers for defining a health status^7^.

Proteomics can have a wide range of application in areas of animal health, production and welfare assessment^8^. Even though it has been limited in clinical application due to many reasons such as cost and lack of genomic data from many species at this point, it is still of promising future to numerous scientific areas^8^. This technology has been used to characterize pathogen-host interaction in salmonella infection of pigs^9^, assess the reproductive health of rams^10^ and search for welfare and stress biomarkers in farm animals^11^. New protein biomarkers in urine have also been identified as a new diagnostic tool for many diseases including babesiosis^12^, chronic kidney disease^13^, and mastitis^14^.

Urinary proteins in animals at different life stages or in different physiological conditions are also wildly studied. Comparison of the urinary protein profiles between cows and heifers has detected the protein changes occurring in pregnancy progresses^15^. Altered urinary proteomes are detected in calves after feeding different amounts of lactose in diet, which indicates calves’ urine a valuable biological material to evaluate renal adjustment to various physiological factors^16^. Urinary protein composition of normal neonatal rats has been compared to adults in one study^17^. The results demonstrate distinct differences correlating with different stages of tissue development, which might provide basis for identification of valuable urinary biomarkers of diseases^17^.

To the best of our knowledge, there has been no published study on urinary proteome of donkeys yet. In the current study, we compared urinary proteins between neonatal donkeys and adult donkeys and hypothesized that there could be specific variations in urinary proteome between the two life stages, which might help identify valuable urinary biomarkers associated with neonatal donkeys’ health state and development.

## Materials and methods

### Samples collection

This study was carried out at a donkey farm in Shandong, China with about 800 Dezhou donkeys in total. Nine male neonatal donkeys aging from 6 to 10 days old and nine adult male donkeys over 3 years old (based on the management record) were involved in this study and were physically healthy according to the medical records. Physical examination was performed for each donkey and no obvious pathological symptoms have been observed. The neonatal donkeys and adult donkeys were having ideal body conditions according to the donkey body condition scoring chart^18^. All adult donkeys were fed the same diet consisted of commercial hay diet and homemade corn-based concentrations. The neonatal donkeys consumed colostrum within 3 h of birth and nursed every 1–2 h for about 5 min each time on the first day of life. They were closely monitored thereafter to confirm the normal nursing frequency and duration. Free flow urine samples were collected mid-stream into sterile containers and were stored on ice temporarily after collection. Upon arriving at the lab, each urine sample was transferred to centrifuge tube and centrifuged at 3000*g* for 15 min at 4 °C. The supernatant was collected by discarding the cellular debrides. To avoid the impact of individual difference on urinary proteins, the nine neonatal urine samples (group N) were randomly assigned into three subgroups with three samples per subgroup. The three urinary samples (10 μL/sample) in each subgroup were pooled to obtain three biological replicants. The adult male donkeys’ urine samples (group A) were processed in the same way as described above. Samples were frozen at − 80 °C until further analysis. All animal procedures were approved by the Animal Care and Use Committee of the China Agricultural University (welfare license No. AW82121202-2-1). Informed consent was obtained before sample collection from the donkey farm. All steps involved in this study were carried out in accordance with the Committee’s approved guidelines and regulations.

### Protein extraction and digestion

Samples were thawed and transferred into lysis buffer (2% SDS, 7 M urea, 1 mg/ml protease inhibitor cocktail (Roche Ltd. Basel, Switzerland)). The homogenated samples were then centrifuged at 3000*g* for 15 min at 4 °C to collect the supernatant. BCA Protein Assay Kit was applied to determine the proteins concentrations. The extracted proteins were finally digested with sequence-grade modified trypsin (Promega, Madison, WI) at 37 °C for 16 h and the peptide mixture was collected.

3 μg peptides dissolved with buffer A (0.1% formic acid in water) was fractionated by high pH separation with Ultimate 3000 system (Thermo Fisher scientific, MA, USA), which was connected to a reverse phase column (4.6 mm × 250 mm, 5 μm, (Waters Corporation, MA, USA)). The column was maintained at 1 ml/min flow rate at 30 °C. Six fractions were collected, and each fraction was dried in a vacuum concentrator for further analysis.

### DIA: nano-high-performance liquid chromatography (HPLC)-tandem mass spectrometry (MS/MS) analysis

The peptides were re-dissolved and analyzed by on-line nanospray LC–MS/MS using EASY-nLC 1200 system with an Orbitrap Fusion Lumos (Thermo Fisher Scientific, MA, USA). The peptide product was loaded onto the analytical column (Acclaim PepMap C18, 75 μm × 25 cm) and then separated with a 120 min gradient (5–35%) buffer B (0.1% formic acid in ACN). The column flow rate was maintained at 200 nl/min with the temperature of 40 °C.

The mass spectrometer was run using a data-dependent method automatically switched between MS and MS/MS mode. The settings are as follows: (1) the mode the automatically switched between MS and MS/MS. (2) MS: scan range (m/z) = 350–1200; AGC target = 1e6; resolution = 120,000; maximum injection time = 50 ms; (3) HCD-MS/MS: AGC target = 1e6; resolution = 30,000; collision energy = 32; stepped CE = 5%. (4) DIA was performed with 60 isolation windows, and each window overlapped 1 m/z.

### Data analysis

Spectronaut X (Biognosys AG, Switzerland) was applied to process and analyze raw data of library-free data-independent acquisition (direct DIA) with default parameters. Spectronaut X was established to search UniprotKB Equus protein sequence database (only reviewed entries, downloaded on 12/20/20 from http://www.uniprot.org). Differentially expressed proteins were analyzed for up- or downregulation using the R statistical computing software (v 3.2.1). Information of protein abundance was collected with at least two valid expression values in each group. Q-value (false discovery rate, FDR) cutoff on precursor and protein level was applied 1%. Quantification was performed for all selected precursors passing the filters. The average top 3 filtered peptides with q value > 1% were used to calculate the major group quantities. Normality of variables was assessed by the Shapiro–Wilk test and student’s t test was performed to filter the differentially expressed proteins with q value < 0.05 and absolute fold change > 1.5.

### Bioinformatics analysis

Analysis of bioinformatics data were conducted using Microsoft Excel, Perseus computational platform, and R statistical computing software^19^. Proteins were annotated against Gene ontology (GO) and Kyoto Encyclopedia of Genes and Genomes (KEGG)^20^ database to evaluate their functions. Significant GO functions and pathways were further examined. The statistically significant level (*P* < 0.05) of differentially expressed proteins involved in GO enrichment analysis were corrected based on Fisher’s exact test.

## Results

### Qualitative and quantitative analysis of urinary proteome in group N and group A

**Figure 1:**
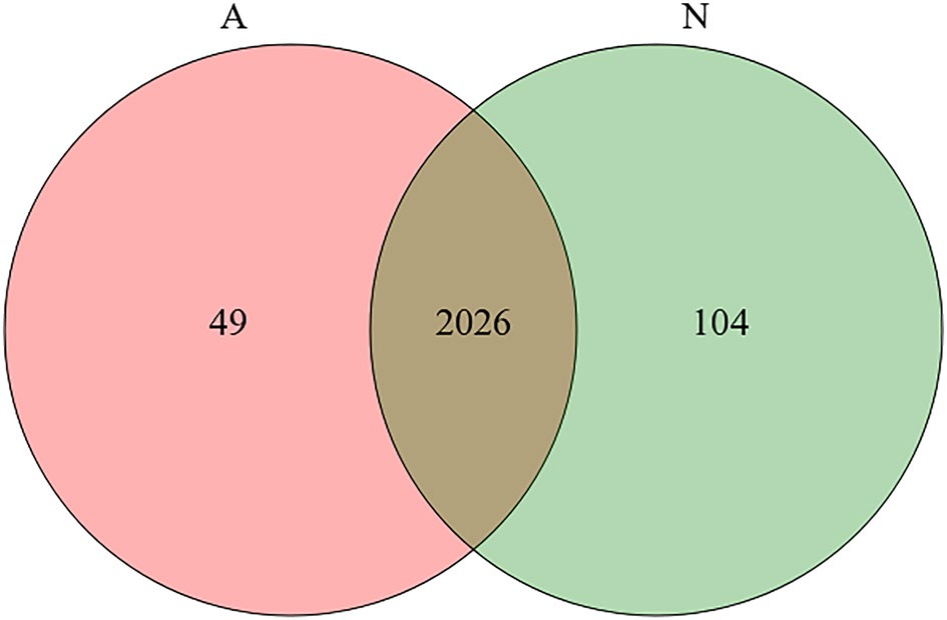
A Venn diagram to compare urinary proteins identified in group N and group A.

We used direct DIA method to establish the profiles of urine protein based on the urinary samples collected from nine neonatal donkeys (group N) and nine adult male donkeys (group A). In total, 2179 proteins were identified in all urine samples. Among the 2179 proteins, 2075 and 2130 proteins were identified in group A and group N, respectively. There were 49 proteins exclusively identified in group A, while 104 unique proteins were uniquely detected in group N (Fig. 1). Proteins involved in neonatal biological and developmental processes included alpha fetoprotein (AFP), peptidase-mitochondrial processing data unit (PMPCB), and upper zone of growth plate and cartilage matrix associated (UCMA) (Supplementary table 1).

### Differentially expressed urinary proteins between the two groups

**Figure 2:**
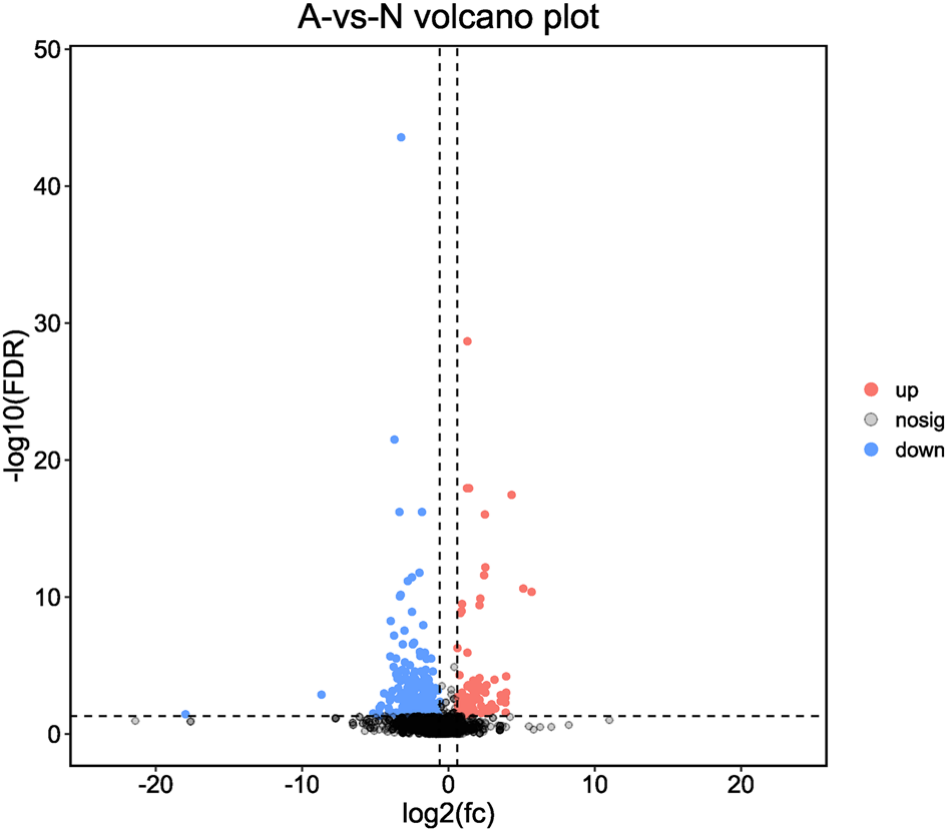
A volcano plot of urinary proteins identified in adult (group A) and neonatal (group N) donkeys. Red dots and blue dots represented upregulated and downregulated proteins in group N compared to proteins in group A, respectively.

Based on the threshold of absolute fold change > 1.5 and *q* < 0.05, we were able to identify 411 differentially expressed urinary proteins between group N and group A. A volcano plot was applied to demonstrate significant differences of these proteins between group N and group A (Fig. 2). Among the most differentially expressed proteins, the levels of cadherin 15 (CDH15) and cartilage intermediate layer protein 2 (CILP2) were higher in donkey foals’ urine, while uromodulin (UMOD) and alpha-1-microglobulin/bikunin precursor (AMBP) were lower compared to adult donkey’s urine (Table 1). Differentially expressed proteins including interleukin-6 receptor (IL6R), gelsolin and polymeric immunoglobulin receptor (PIGR) were significantly more expressed in group A than in group N and were involved in immune pathways (Table 1, Supplementary table 2). Galactosidase beta 1 (GLB1) and hexosaminidase subunit beta (HEXB) were both more abundant in group A and involved in pathways of glycosaminoglycan degradation and glycosphingolipid biosynthesis (Table 1, Supplementary Table 2).

**Table 1:** Differentially expressed proteins in urine of adult (group A) compared to neonatal (group N) donkeys (A vs N) mentioned in this manuscript.

Protein names	Symbols	FC^a^	*P* value
Uromodulin	UMOD	− 3.234	2.52E−47
Cartilage intermediate layer protein 2	CILP2	2.498	9.35E−17
Alpha-1-microglobulin/bikunin prcursor	AMBP	− 2.499	4.21E−14
Cadherin 15	CDH15	5.105	3.17E−13
Interleukin 6 receptor	IL6R	− 3.042	0.0047
Gelsolin	GSN	− 1.058	1.17E−06
polymeric immunoglobulin receptor	PIGR	− 2.803	3.69E−05
Galactosidase beta 1	GLB1	− 1.376	0.0001
Hexosaminidase subunit beta	HEXB	− 2.170	0.0003
Leucine rich repeat containing 15	LRRC15	4.321	1.93E−20
Colony stimulating factor 1 receptor	CSF1R	2.123	4.44E−06
Plasminogen	PLG	1.758	0.0001

^a^FC, fold change, a measure used to quantify the changes between urinary proteins in group N and group A. If the fold change value > 1.5, the number of proteins in group N is higher than in group A. if fold change value < 0.67, the number of proteins in group N is less than in group A.

### GO enrichment analysis of the differentially expressed urinary proteins between the two groups

**Figure 3:**
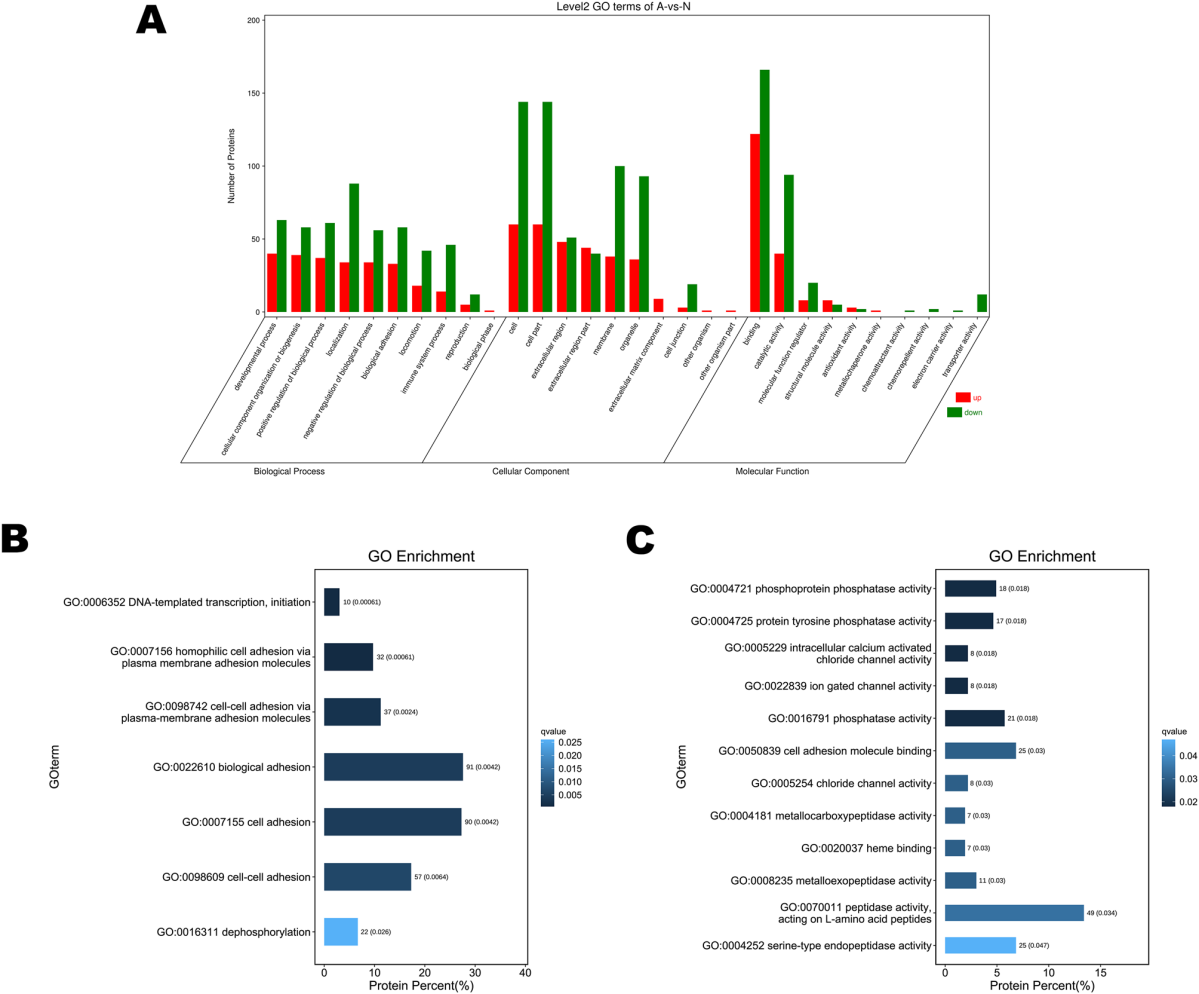
Functional analysis of urinary proteins between group N and group A. (**A**) GO enrichment analysis of differentially expressed proteins identified in urine samples of neonatal donkeys and adult donkeys. Proteins were divided into three categories including biological process, cellular component, and molecular function. (**B**) The most differentiated activities in biological process category identified between group N and group A. (**C**). The most differentiated activities in Metabolic function category identified between group N and group A.

GO enrichment analysis was used to compare biological functions of differentially expressed urinary proteins between neonatal donkeys and adult donkeys. Three biological functional sets namely biological process (BP), cellular component (CC) and molecular function (MF) were established to classify these proteins (Fig. 3A). The most prevalent BPs in both groups were cellular process, single-organism process, metabolic process and biological regulation. Binding and catalytic activities were the two most enriched biological functions in MF category, while cell and cell part were most enriched in CCs. The most differentiated BPs between the two groups were DNA-templated transcription, biological adhesion, and cell adhesion (Fig. 3B). Leucine rich repeat containing 15 (LRRC15) and colony stimulating factor 1 receptor (CSF1R) were mainly involved in BP and expressed significantly higher in the urine of neonatal donkeys (Table 1, Supplementary table 3). The most significantly different MFs between the two groups according to GO annotation included phosphatase activity and ion gated channel (Fig. 3C).

### KEGG pathway analysis of urinary proteins in group N and group A

**Figure 4:**
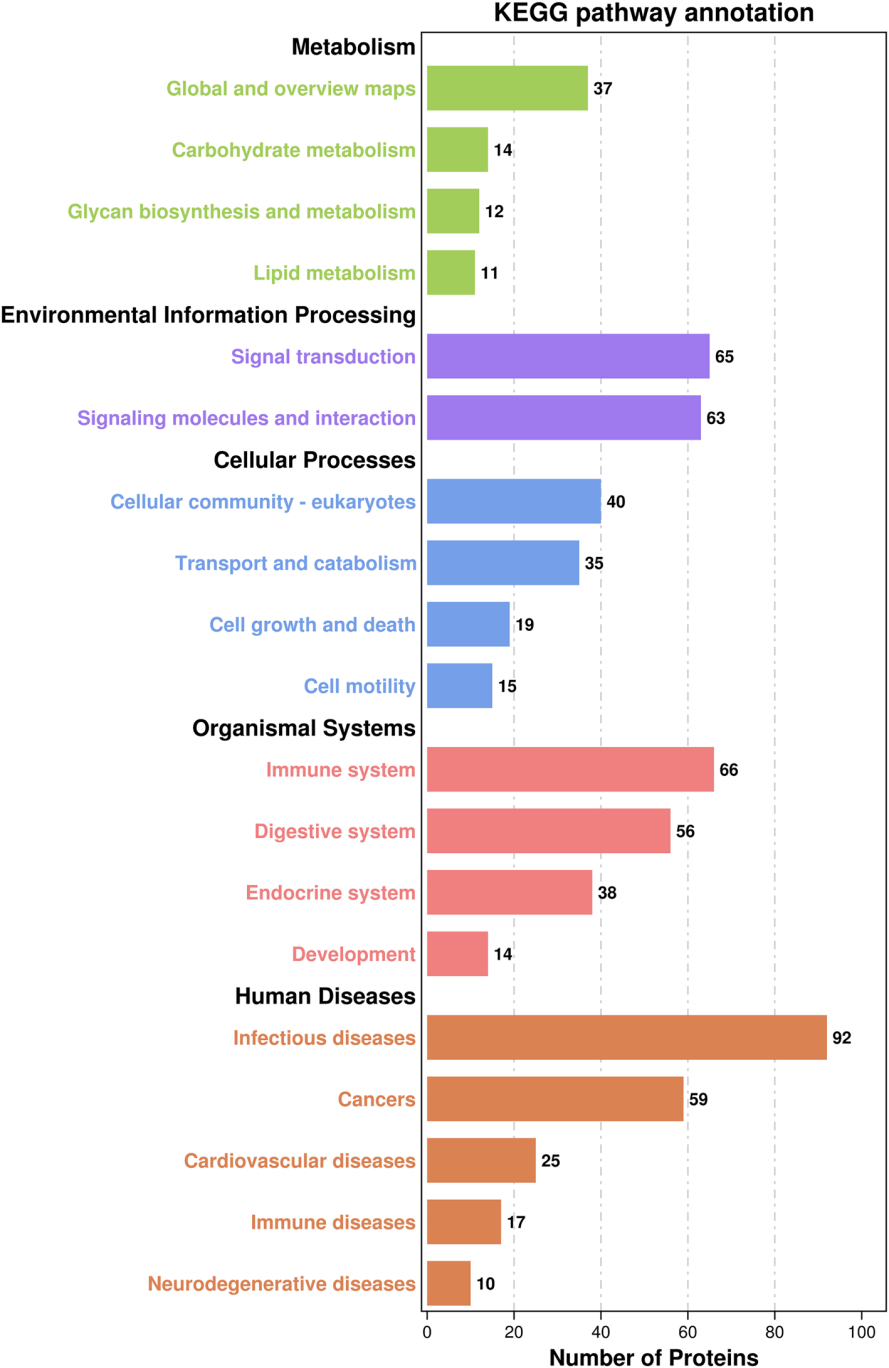
The KEGG pathway annotation and categorization^21^ of the identified urinary proteins from proteome database of donkeys.

The 411 most differentially expressed proteins were related to 146 KEGG pathways. At KEGG pathway level, infectious diseases, immune system, signal transduction, and signaling molecules and interaction were the most prevalent pathways^21^ (Fig. 4). Plasminogen (PLG) was one of the proteins involved in infectious disease pathway and had higher expression in group N (Table 1, Supplementary table 2).

## Discussion

Urine is formed via plasma filtration by glomeruli in the kidney and associated with systemic as well as local physiology^22^. The composition of urine is less complex in comparison with plasma or serum, making it more popular to mass spectrometric analysis^3^. Urinary proteome, therefore, can be used as a potential diagnostic tool for the analysis of systemic functions in humans and other species^7,12,13^. To the best of our knowledge, this is the first comparative study of urinary proteins in donkeys. It has been shown that sex might impact the composition of urinary proteins^5^, hence, we only involved male donkeys in the study to minimize the sex bias. Investigation to compare urinary proteomes between male and female donkeys will be helpful to provide more insight in the urinary proteome of donkeys. In the current study, we investigated qualitative and quantitative characteristics of urinary proteome of male donkeys at two different life stages and demonstrated significant difference of urinary proteins between the two groups.

The DIA technology is independent of the composition of precursor ions but capable of coupling with targeted data analysis for protein analysis^23^. This technique is sensitive at peptide as well as protein detecting and also able to identify some novel peptides, which allows more accurate proteome analysis^24^. Direct DIA analysis achieves higher protein quantification over traditional data-dependent analysis (DDA) and eliminates bias against the reproductive quantification of low-abundant proteins^25^. Even though direct DIA detects less protein groups than deep DIA method^26^, it still produces relatively high enough quantification of proteins in complex mixtures^25^.

So far, there is only one study investigating the urinary protein profiles from a 12-year-old quarter horse via LC–MS/MS DDA^6^. UMOD, CHD15, AMBP and CILP2 are identified in that study and considered common to the urinary tract and kidneys^6^. In the current study, the four urinary proteins were detected in both groups of donkeys, which were consistent with the result in horses. UMOD and CHD have also been reported in neonatal calves which were associated with renal tubules function and cardiovascular system development, respectively^7^. However, the different expression level of these urinary proteins between neonatal and adult donkeys requires further exploration for their roles in different developmental stages.

Several proteins were exclusively identified in urine of neonatal donkeys. AFP, one of the unique neonatal urinary proteins in the current study, is produced by fetal liver and has similar physical and chemical characteristics of albumin in mammalian species^27^. Even though its biological role is not completely understood, it has been considered as a critical diagnostic biomarker especially for neonatal diseases, for example, neonatal hepatitis^28^. AFP has also been detected in bone material of neonatal dogs^29^, while in this study, was identified in urine of neonatal donkeys. As an important diagnostic protein candidate in neonatal donkeys, it would will be helpful to measure AFP concentrations and establish a normal reference value as a baseline of healthy newborn donkeys. PMPCB is the main peptidase responsible for mitochondrial protein processing^30^ and has been associated with mammalian mitochondrial stress responses^31^. PMPCB mutation is identified as a new mechanism in mitochondrial diseases including neurodegeneration in young children^32^, which indicates its association with some neonatal diseases. UCMA is associated with the development of articular chondrocytes and plays a critical role in chondrocytes expression^33^. Upregulation of UCMA is detected in neonatal cartilage^34^ and growing mouse limb joints^35^. It is the first time of this protein to be identified in equine. Further investigation will be helpful for understanding the association between cartilage growing status and the expression of UCMA in equine species. These exclusively expressed proteins in neonatal donkeys, which have been proved crucial in neonatal development in other species, might be novel biomarker candidates for monitoring physiological state in neonatal donkeys.

IL6R, gelsolin and PIGR were categorized to immune system pathways in this study including Fc gamma R-medicated phagocytosis, IgA production in intestinal immune network, and hematopoietic cell lineage. Neonates possess a developing immune system, which renders newborns highly susceptible to environmental insults^36^. Significantly less expressed proteins associated with immunity between the adult and neonatal donkeys might provide evidence of higher infection risk in neonatal donkeys. IL-6, as a proinflammatory cytokine, is elevated systemically in the acute phase response^37^. IL-6 is necessary to activate Stat3 transcription factor which plays an important role in antimicrobial response to urinary tract infection (UTI)^38^. Deficiency in IL-6 and downregulation of IL6R has been associated with increased risk of renal bacterial infection^39,40^. In this study, higher level of IL6R was detected in urine of adult donkeys than in neonatal donkeys, which might indicate the higher vulnerability of urinary tract infection in neonatal donkeys compared to adult donkeys. Its biological role of immunity regulation in neonatal donkeys required further investigation.

Gelsolin is another immune-related protein that had significant higher expression in adult donkeys than in neonatal donkeys. Gelsolin is involved in immune system as an anti-inflammatory modulator and its depletion can lead to damage in the immune cells^41^. There is evidence that it could trigger an anti-inflammatory response and modify migration of cells to reduce the inflammatory reaction^42,43^. The PIGR is responsible for transporting mucosal IgA into the lumen of organs^44^. Urinary PIGR has been detected decreased significantly in systemic *Escherichia coli* infection in mice^45^. Lower level of immune-related urinary proteins in neonatal donkeys might be associated with the more vulnerable immune system compared to adult donkeys. The present study provided a basic reference that healthy neonatal donkey foals had lower abundance of these proteins. Further studies exploring the changes of these proteins in healthy and diseased neonatal donkeys may facilitate understanding their immune response against infectious diseases.

GLB1 and HEXB were both detected at significantly higher levels in adult donkey than in neonatal donkeys. They were both involved in pathways of glycosaminoglycan degradation and glycosphingolipid biosynthesis. In one study, GLB1 are significantly higher expressed in bone marrow mesenchymal stem cells recovered from geriatric horses than newborn foals^46^, which is consistent with the results in our study. There is evidence that GLB1 is a senescence marker with higher gene expression in senescent cells^47^. It may explain the higher level of GLB1 in adult than in neonatal donkeys. HEXB is an enzyme generally present in the lysosome. It is found essential for glycoprotein metabolism and the maintenance of cell homeostasis in mammals^48^. More functions of HEXB have been studied, for example, it is crucial for defense against bacterial invasion^49^. Besides its role in immune system, HEXB is found stably expressed in microglia and has been used as an important marker to study central nervous system diseases^50^. In horses, it has been found significantly higher expressed in chondrocytes of individuals (1–4 years old) with osteochondrosis^51^ due to its participation in cartilage turnover^52^. Since HEXB are associated with several diseases, further investigation on the absolute expression in adult and neonatal donkeys could help establish a basic reference for its expression and monitor for diseases in donkeys.

The function of differentially expressed proteins in urine of neonatal donkeys and adult donkeys has been categorized by GO analysis. The most significantly different BP involving the most differentially expressed proteins were biological adhesion and cell adhesion. LRRC15 was the protein involved in biological adhesion and expressed significantly higher in neonatal donkeys. LRRC15 is a member of LRR superfamily which participates in cell adhesion, signal transduction and DNA repair by working on protein–protein and protein–matrix interaction^53,54^. It has been shown that LRRC15 was abundant in mineralized tissues^55^. Further research has illustrated that LRRC15 was able to positively regulate osteogenic differentiation^56^, which might explain its higher expression in donkey foal’s urinary proteins since young animals tend to have more active bone growth. In the current study, it is the first time to report the presence of LRRC15 in donkey urinary proteins and its potential as a biological marker associated with osteogenic differentiation might be worth further investigation in neonatal donkeys. Other studies also find that some urinary proteins are commonly involved in immune response since urinary tract is under constant threat of infection^57,58^. CSF1R was a protein expressed significantly higher in neonatal donkeys in this study and involved in some significantly differentiated BPs such as regulation of actin-filament and cell morphogenesis based on GO analysis. CSF1R plays a critical role in signaling in mononuclear phagocyte system and macrophages^59^. In particular, it modulates migration, differentiation and proliferation of macrophages^60^. High expression in urine samples of healthy neonatal donkeys might infer its importance in neonatal immune system regulation.

According to the results of KEGG pathway annotation, infectious disease is one of the most prevalent pathways. Among the infectious disease KEGG class, influenza A was the most differentiated pathways and PLG was one of the most significantly differentially expressed proteins in the pathway. PLG is abundant in plasma and essential to fibrinolytic system that maintain a coagulation balance with procoagulative activities^61^. It is also a precursor that can be activated by plasminogen activator as active plasmin^62^. Plasminogen-plasmin system is involved in many critical physiological and pathologic processes including cell migration, autoimmune, tumor formation and neurodegeneration^63^. In the current study, it was found a significant higher expression in urine of neonatal donkeys, and it could be a critical component in neonatal immune system.

## Conclusions

In summary, this is the first study to examine donkey urinary proteomics and compare the composition and function of urinary proteins in neonatal and adult donkeys. AFP, PMPCB and UCMA would require further study to explore their potential in monitoring health status of neonatal donkeys. The differentiated expression profiles revealed a couple of immune-related proteins including IL6R, gelsolin and PIGR, which might facilitate understanding immune system development of neonatal donkeys. A couple of immune-related activities or pathways were detected in both GO and KEGG analysis, which might help with the selection of potential urinary protein biomarkers to monitor the immune system of neonatal donkeys.

## Supplementary Information

Supplementary Legends.

Supplementary Table 1.

Supplementary Table 2.

Supplementary Table 3.

## Data Availability

Deposited proteomics data can be accessed with username: reviewer_pxd031613@ebi.ac.uk and password: D5N5AxwV at website http://www.ebi.ac.uk/pride. The other data related to this study could be accessed via Figshare at https://figshare.com/s/825860c171cba15dd2e1. The DOI of the supplementary data at Figshare is 10.6084/m9.figshare.19070174.
