# Supplementary material for: DIA mass spectrometry characterizes urinary proteomics in neonatal and adult donkeys
Source: Sci Rep. 2022 Dec 30;12:22590. doi: 10.1038/s41598-022-27245-0 (PMC9803668; doi:10.1038/s41598-022-27245-0)
Supplement: Supplementary file 1 — Supplementary Legends. [file 41598_2022_27245_MOESM1_ESM.docx]

**Supplementary table 1**. A list of exclusively expressed proteins in the group N.
**Supplementary table 2**. A list of KEGG pathways of differentially expressed proteins of the two groups.

**Supplementary table 3**. A list of top ten GO annotations of BPs, MFs and CCs of differentially expressed proteins identified in the two groups.
